# Sodium-myoinositol cotransporter-1, SMIT1, mediates the production of reactive oxygen species induced by hyperglycemia in the heart

**DOI:** 10.1038/srep41166

**Published:** 2017-01-27

**Authors:** Anne Van Steenbergen, Magali Balteau, Audrey Ginion, Laura Ferté, Sylvain Battault, Christophe de Meester de Ravenstein, Jean-Luc Balligand, Evangelos-Panagiotis Daskalopoulos, Patrick Gilon, Florin Despa, Sanda Despa, Jean-Louis Vanoverschelde, Sandrine Horman, Hermann Koepsell, Gerard Berry, Louis Hue, Luc Bertrand, Christophe Beauloye

**Affiliations:** 1 Université catholique de Louvain, Institut de Recherche Expérimentale et Clinique, Pôle de Recherche Cardiovasculaire, Brussels, Belgium; 2 Université catholique de Louvain, Institut de Recherche Expérimentale et Clinique, Pole of Pharmacology and Therapeutics, Brussels, Belgium; 3 Cliniques Universitaires Saint-Luc, Department of Medicine, Brussels, Belgium; 4 Université Catholique de Louvain, Institut de Recherche Expérimentale et Clinique, Pôle d’Endocrinologie, Diabète et Nutrition, Brussels, Belgium; 5 University of Kentucky, Department of Pharmacology and Nutritional Sciences, Lexington, KY, USA; 6 Cliniques Universitaires Saint Luc, Division of Cardiology, Brussels, Belgium; 7 University of Würzburg, Department of Molecular Plant Physiology and Biophysics, Julius von Sachs Institute, Würzburg, Germany; 8 Harvard Medical School, Children’s Hospital Boston, Division of Genetics and Genomics, Department of Pediatrics, Boston, MA, USA; 9 Université catholique de Louvain, de Duve Institute, Brussels, Belgium

## Abstract

Hyperglycemia (HG) stimulates the production of reactive oxygen species in the heart through activation of NADPH oxidase 2 (NOX2). This production is independent of glucose metabolism but requires sodium/glucose cotransporters (SGLT). Seven SGLT isoforms (SGLT1 to 6 and sodium-myoinositol cotransporter-1, SMIT1) are known, although their expression and function in the heart remain elusive. We investigated these 7 isoforms and found that only SGLT1 and SMIT1 were expressed in mouse, rat and human hearts. In cardiomyocytes, galactose (transported through SGLT1) did not activate NOX2. Accordingly, SGLT1 deficiency did not prevent HG-induced NOX2 activation, ruling it out in the cellular response to HG. In contrast, myo-inositol (transported through SMIT1) reproduced the toxic effects of HG. SMIT1 overexpression exacerbated glucotoxicity and sensitized cardiomyocytes to HG, whereas its deletion prevented HG-induced NOX2 activation. In conclusion, our results show that heart SMIT1 senses HG and triggers NOX2 activation. This could participate in the redox signaling in hyperglycemic heart and contribute to the pathophysiology of diabetic cardiomyopathy.

Diabetes and hyperglycemia (HG) are major risk factors for cardiovascular diseases^1^,^2^. HG is also associated with adverse outcomes and increased mortality in patients with acute myocardial infarction, even in the absence of diabetes^3^,^4^,^5^. Prolonged exposure of cardiomyocytes to HG indeed has deleterious effects, such as alterations of myofibrillar structure and intercellular connections^6^, possibly resulting in cell death^7^,^8^,^9^. Several hypotheses have been proposed to explain the mechanism of such glucotoxicity. A major one is the exacerbated production of reactive oxygen species (ROS) triggered by HG^10^. We have demonstrated previously that HG induces NADPH oxidase 2 (NOX2) activation (the major isoform of NOX in the heart), leading to ROS production independently of glucose metabolism^11^. Actually, HG-induced ROS production depends on sodium/glucose co-transporters (SGLT), which typically use the downhill sodium gradient to drive sugar uptake, but not on classical facilitated-diffusion glucose transporters^11^,^12^. Indeed, phlorizin, a SGLT family-specific inhibitor, prevents HG-induced ROS production. Furthermore, α-methyl-D-glucopyranoside (αMG), a non-metabolizable glucose analogue, which is transported through SGLT, but not GLUT, mimics HG-induced ROS production in adult rat cardiomyocytes. On the other hand, 2-deoxyglucose (2DG), a glucose analogue with low affinity for SGLT but high affinity for GLUT, is unable to reproduce HG-induced ROS production^11^.

Seven SGLT isoforms have been described so far^13^:

- SGLT1 (encoded by *SLC5a1)* is mainly expressed in the brush border membranes of enterocytes and in proximal tubules of the kidneys. It is responsible for active glucose absorption in both tissues^14^.
- SGLT2 *(SLC5a2)* is mainly expressed in the kidneys, playing a major role in glucose reabsorption in tubules^15^,^16^.
- SGLT3 *(SLC5a4)* has been regarded as a glucose “sensor” rather than a co-transporter^17^. Compared to SGLT1 and SGLT2, its affinity for glucose is rather low (Km around = 20 mM), and the binding of sugar to human SGLT3 triggers membrane depolarization without any sugar transport. 2 genes are known in rodents (mice and rats): *SLC5a4a* coding for SGLT3a, and *SLC5a4b* coding for SGLT3b^18^. Sugar-induced current mediated by SGLT3a occurs only at acidic pH^19^. In contrast to human SGLT3, rodent SGLT3b transports sugar^20^.
- Little is known about SGLT4 *(SLC5a8)*, except that it is a widely-expressed mannose transporter^21^.
- SGLT5 *(SLC5a9)* is exclusively expressed in the kidneys where it reabsorbs glucose and galactose^22^.
- SMIT1 and SGLT6 (also called SMIT2) are encoded by *SLC5a3* and *SLC5a11*, respectively. They are expressed in the brain and kidneys where they co-transport myo-inositol with sodium^23^,^24^. Interestingly, their affinity for glucose is rather low^15^,^25^.

The tissue expression of SGLT1 and SGLT2 has been studied extensively, including in the heart. SGLT1 is known to be highly expressed in the heart^26^,^27^, even if its function in this organ remains poorly understood. There are conflicting reports about SGLT2 expression in cardiac tissues^16^,^21^,^26^,^28^,^29^, and little is known about the expression of other SGLT isoforms in the heart. To investigate the SGLT isoform involved in HG-induced ROS production, we studied the expression profile of the 7 SGLT isoforms in murine, rat and human hearts. We found that 2 SGLT isoforms, SGLT1 and SMIT1, are expressed in the heart and cardiomyocytes. We demonstrated, for the first time, that SMIT1 confers to cardiomyocytes the ability to detect HG and evokes NOX2 activation as well as ROS production.

## Results

### SGLT1 and SMIT1 are SGLT isoforms mainly expressed in the heart and cardiomyocytes

**Figure 1:**
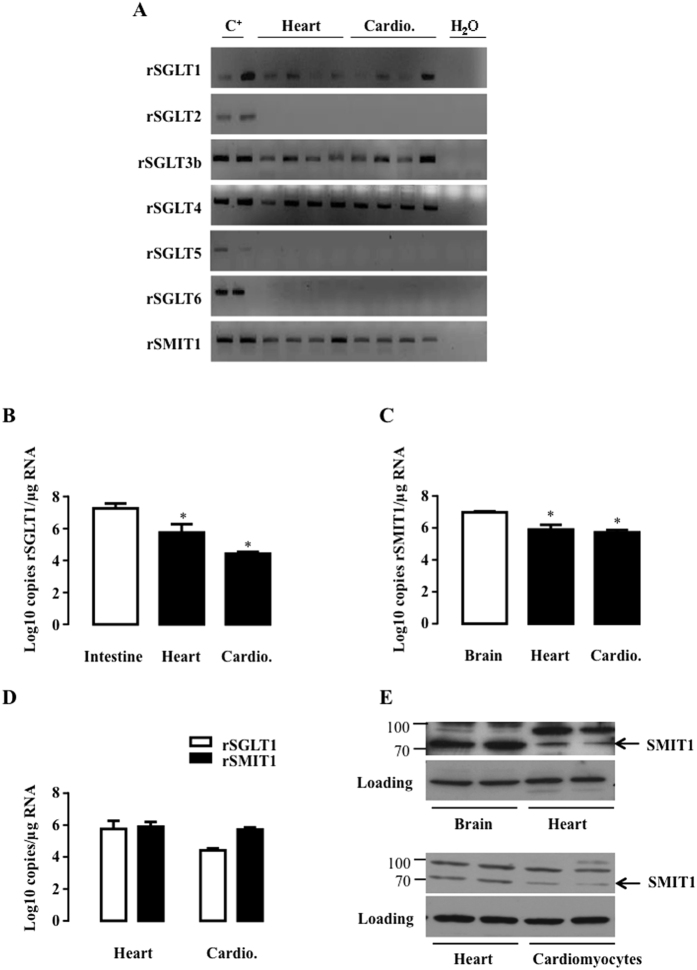
Detection of SGLT isoforms in rat heart and cardiomyocytes. (**A**) SGLT1, SGLT2, SGLT3b, SGLT4, SGLT5, SGLT6 and SMIT1 detection by RT-PCR and ethidium bromide-stained agarose gels on mRNA extracted from hearts (n = 4) and isolated cardiomyocytes (cardio. n = 4) of rats. Positive controls were intestine for SGLT1, kidney for SGLT2, SGLT3, SGLT4 and SGLT5 and brain for SGLT6 and SMIT1. mRNA copy number per μg of RNA of SGLT1 (**B**) and SMIT1 (**C**) were measured in rat hearts (n = 4) and cardiomyocytes (n = 4) and compared to a positive control (n = 3). Data are means ± SEM. Statistical analysis was by one-way ANOVA. *Indicates values statistically different from corresponding control tissue, p ≤ 0.05. (**D**) Comparison of SGLT1 and SMIT1 mRNA copy numbers/μg of RNA between hearts (n = 4) and cardiomyocytes (n = 4). Data were normalized to hypoxanthine guanine phosphoribosyl transferase (HPRT1) and expressed as Log10 copy numbers/μg RNA. (**E**) SMIT1 protein expression in rat heart compared to rat brain and in isolated rat cardiomyocytes in culture compared to total heart extract. eEF-2 detection is used as loading control. Full-length blots are presented in Supplementary Figure 4.

**Figure 2:**
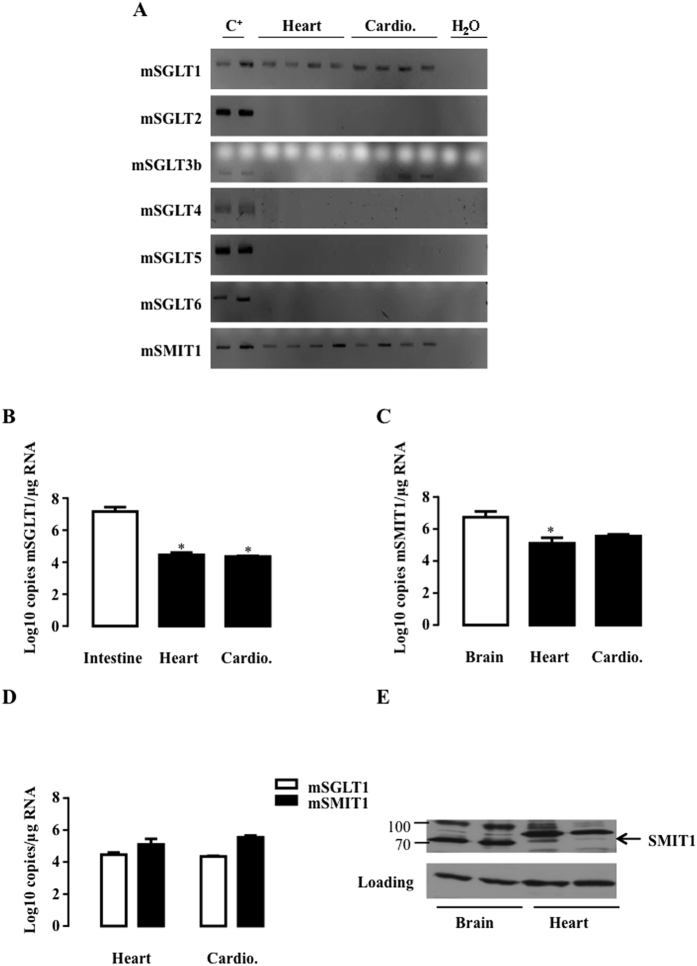
Detection of SGLT isoforms in mouse heart and cardiomyocytes. (**A**) SGLT1, SGLT2, SGLT3b, SGLT4, SGLT5, SGLT6 and SMIT1 detection by RT-PCR and ethidium bromide-stained agarose gels on mRNA extracted from hearts (n = 4) and isolated cardiomyocytes (cardio. n = 4) of mice. Positive controls were intestine for SGLT1, kidney for SGLT2, SGLT3, SGLT4 and SGLT5 and brain for SGLT6 and SMIT1. mRNA copy number per μg of RNA of SGLT1 (**B**) and SMIT1 (**C**) were measured in mice hearts (n = 4) and cardiomyocytes (n = 4) and compared to a positive control (n = 3). Data are means ± SEM. Statistical analysis was by one-way ANOVA. *Indicates values statistically different from corresponding control tissue, p ≤ 0.05. (**D**) Comparison of SGLT1 and SMIT1 mRNA copy numbers/μg of RNA between hearts (n = 4) and cardiomyocytes (n = 4). Data were normalized to ribosomal protein L32 (RPL32) and expressed as Log10 copy numbers/μg RNA. (**E**) SMIT1 protein expression in murine heart compared to murine brain. eEF-2 detection is used as loading control. Full-length blots are presented in Supplementary Figure 5.

**Figure 3:**
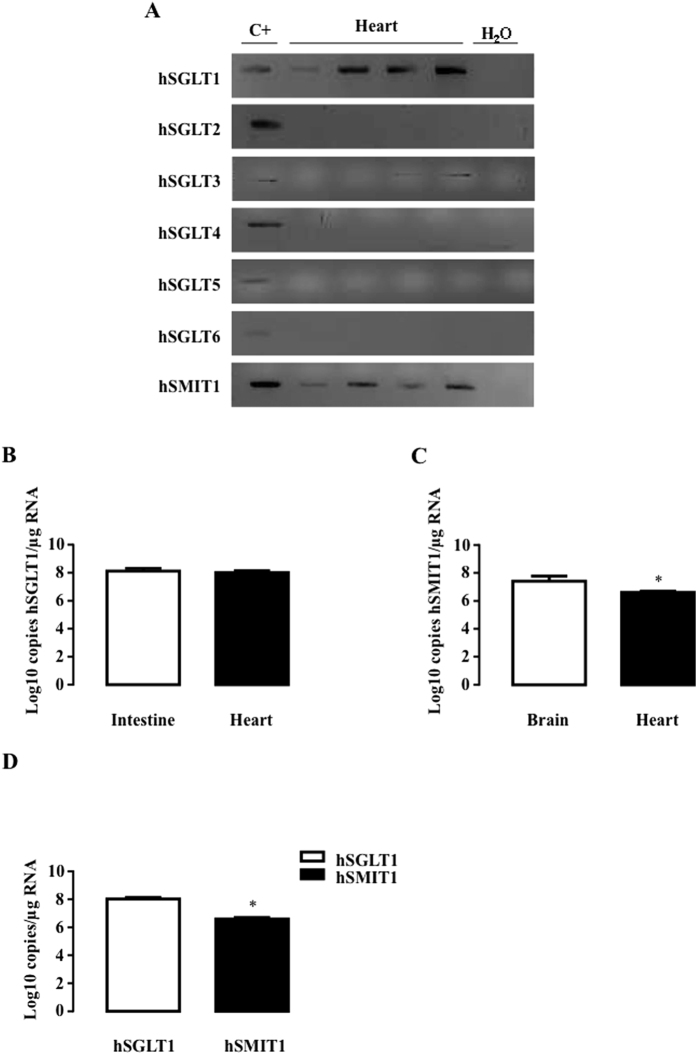
Detection of SGLT isoforms in human hearts. (**A**) SGLT1, SGLT2, SGLT3, SGLT4, SGLT5, SGLT6 and SMIT1 detection by RT-PCR and ethidium bromide-stained agarose gels on mRNA extracted from non-failing human hearts (n = 4). Positive controls were intestine for SGLT1, kidney for SGLT2, SGLT3, SGLT4 and SGLT5, and brain for SGLT6 and SMIT1. mRNA copy numbers/μg of SGLT1 (**B**) and SMIT1 (**C**) RNA were measured in non-failing human hearts (n = 7) and compared to a positive control (n = 3). The clinical characteristics of patients are presented in Supplementary Table 1. (**D**) Comparison of SGLT1 and SMIT1 mRNA copy numbers/μg of RNA in human hearts (n = 7). Data were normalized to RPL32 and expressed as Log10 copy numbers/μg RNA. Data are means ± SEM. Statistical analysis was by Student’s t-test. *Indicates values statistically different from (**C**) corresponding control tissue (**D**) hSGLT1 mRNA expression, p ≤ 0.05.

We firstly investigated the expression of all SGLT isoforms (SGLT1 to 6 and SMIT1) in the heart and purified cardiomyocytes of rats (Fig. 1) and mice (Fig. 2) and in human hearts (Fig. 3). Because of the poor specificity of commercially-available antibodies for detecting the different isoforms^26^,^30^, we resorted to polymerase chain reaction (PCR) to specifically detect all the known isoforms. As expected, SGLT1 was expressed in the heart and cardiomyocytes of adult rats and mice as well as in the human heart. SGLT2, SGLT5 and SGLT6 were undetectable in all species (Fig. 1A, 2A and 3A). SGLT3 and SGLT4 were marginally expressed and became detectable in rats only through nested PCR, as proposed by O’Malley *et al*.^31^. SGLT3(b) was barely present in mice and humans, and SGLT4 was not apparent. In contrast, SMIT1 - like SGLT1 - was readily detectable in all species. SGLT1 and SMIT1 were then quantified and compared to their expression in positive control tissue (intestine for SGLT1 and brain for SMIT1) by quantitative-PCR (qRT-PCR). Compared to the intestine, SGLT1 mRNA was less expressed in the hearts of rats (Fig. 1B) and mice (Fig. 2B), but equally expressed in human hearts (Fig. 3B), in agreement with earlier findings^26^. SMIT1 mRNA expression was about 10-fold lower in the heart than in the brain of all species (Fig. 1C, 2C and 3C). Even if they were less expressed than in their respective bona-fide tissues, SMIT1 and SGLT1 mRNA levels were comparable in the heart as well as in cardiomyocytes from rats (Fig. 1D) and mice (Fig. 2D). Human hearts exhibited higher SGLT1 compared to SMIT1 mRNA levels (Fig. 3D). Even if antibody specificity could be questioned, SMIT1 protein seemed to be detected by immunoblotting in rat (Fig. 1E) and mouse hearts (Fig. 2E). We confirmed that SMIT1 protein is also present in isolated cardiomyocytes in culture (Fig. 1E). In summary, SGLT1 and SMIT1 are SGLT isoforms significantly expressed in the heart.

### Myo-inositol, transported through SMIT1, mimics HG-induced NOX2 activation and ROS production

**Figure 4:**
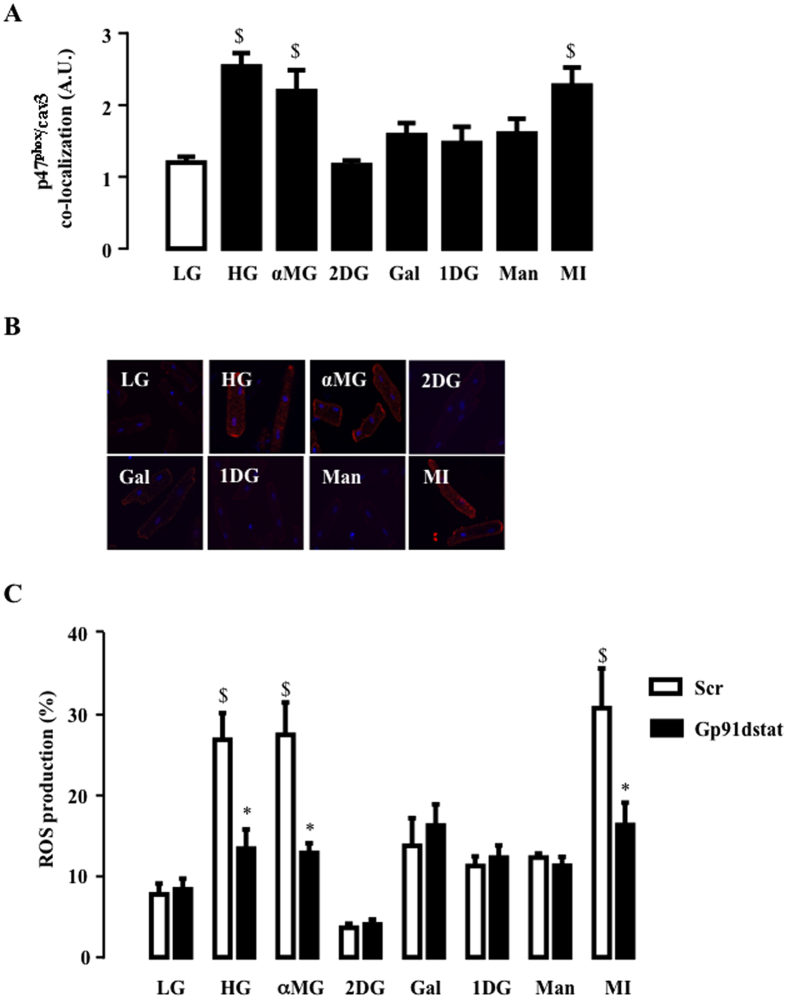
Effect of glucose analogues on NOX2 activation and ROS production. (**A**) Effect of 5 mM (LG) or 16 mM of glucose (HG), α-Methyl-D-glucose (αMG), 2-deoxy-glucose (2DG), galactose (Gal), 1-deoxy-glucose (1DG), mannose (Man) or myo-inositol (MI) (under 5 mM glucose background) on HG-induced p47phox co-localization close to cav3. The close proximity between p47phox and cav3, as detected by PLA, was assessed 90 min after exposure to glucose analogues. Typical pictures of the effect of glucose analogues are shown in (**B**). White lines correspond to 20 μm. (**C**) ROS production measured 2 h after LG, HG, αMG, 2DG, Gal, 1DG, Man and MI. 2.5 μM of Gp91dstat or scrambled peptide were added 15 min before glucose analogues. The data are means ± SEM, (n = 4). Statistical analysis was by (**A**) one-way ANOVA and (**C**) two-way ANOVA. ^$^Indicates values statistically different from LG, p ≤ 0.05. *Indicates values statistically different from the corresponding HG sample without treatment, p ≤ 0.05.

Electrophysiological studies demonstrated high affinity of SGLT1 for galactose^32^, SGLT3(b) for 1-deoxy-glucose (1DG)^20^, SGLT4 for mannose^21^ and SMIT1 for myo-inositol^33^. We took advantage of this substrate specificity to ascertain which SGLT isoform could be involved in the HG response. Adult rat cardiomyocytes were exposed to these glucose analogues to test their capacity to activate NOX2 and to stimulate ROS production. NOX2 activation was evaluated by measuring the translocation of p47^phox^ (a NOX2-activating subunit) close to caveolin-3 (cav3) and ROS production. αMG (transported through all SGLTs) induced NOX2 activation, as already described^34^, but 2DG (transported through GLUT), 1DG (SGLT3) and mannose (SGLT4) did not. Interestingly, myo-inositiol triggered translocation of p47^phox^ close to cav3 (Fig. 4A,B) and increased ROS production (Fig. 4C), thereby mimicking the toxicity induced by HG. Furthermore, Gp91dstat, a specific NOX2 inhibitor, prevented myo-inositol-induced ROS production (Fig. 4C). Finally, exposure of cardiomyocytes to galactose did not mimic the effect of HG or MI, indicating that SGLT1 does not mediate HG-induced ROS production. Taken together, our data suggest that SMIT1 is the SGLT isoform mainly responsible for the detection of increased glucose concentration, leading to ROS production.

### SGLT1 does not mediate HG-induced NOX2 activation

**Figure 5:**
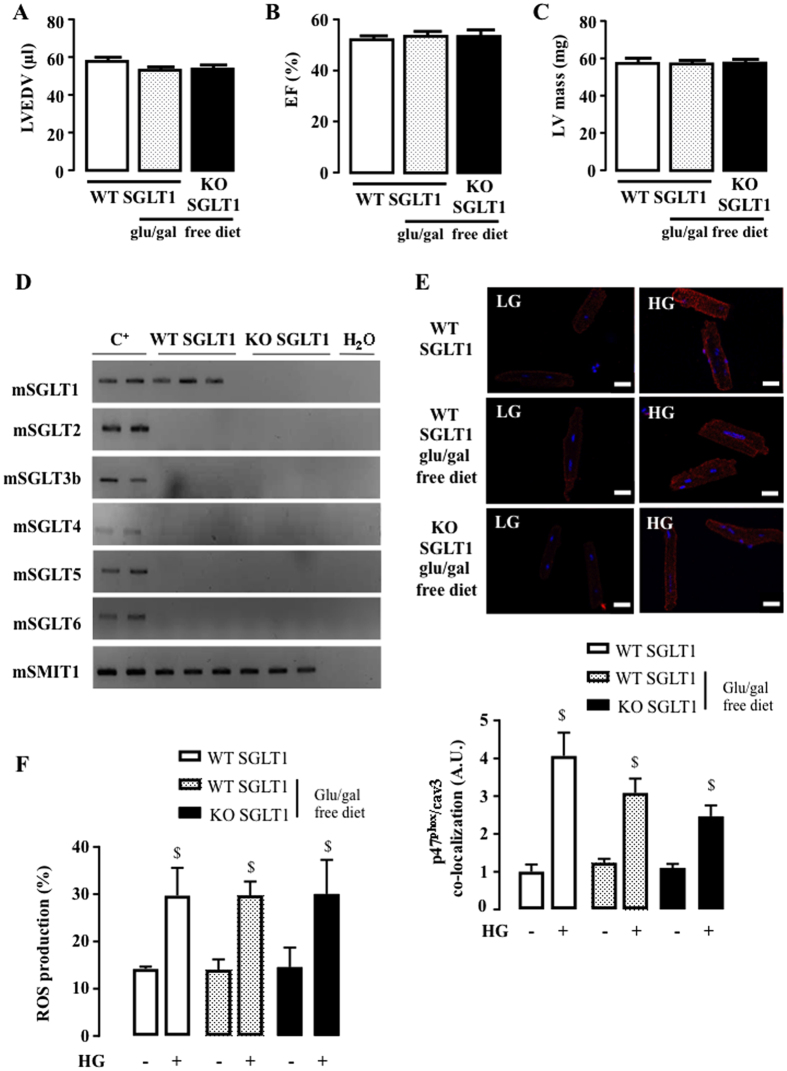
Impact of SGLT1 deletion on HG-induced NOX2 activation and ROS production. (**A**) LVEDV, (**B**) EF and (**C**) LV mass were measured by echocardiography of SGLT1 WT under usual diet (n = 10), of SGLT1 WT submitted to glucose and galactose free diet (glu/gal free diet, n = 10) and of SGLT1 KO (n = 10) mice. Echocardiographic data in M-mode and 2D parasternal long axis are presented in Supplementary Table 4. (**D**) Detection of SGLT1, SGLT2, SGLT3b, SGLT4, SGLT5, SGLT6 and SMIT1 by RT-PCR and ethidium bromide-stained agarose gels on mRNA extracted from the hearts of SGLT1 KO mice (n = 3) compared to WT mice (n = 3). Positive controls were intestine for SGLT1, kidneys for SGLT2, SGLT3, SGLT4 and SGLT5, brain for SGLT6 and SMIT1. (**E**) Quantification of HG-induced p47phox translocation close to cav3 in SGLT1 WT mice (with and without glu/gal free diet) compared to SGLT1 KO mice. Adult mouse cardiomyocytes were isolated from SGLT1 WT (n = 7), SGLT1 WT submitted to glu/gal free diet (n = 7) and SGLT1 KO (n = 7) hearts. PLA was performed 90 min after stimulation with HG and compared to LG. White lines correspond to 20 μm. (**F**) ROS production induced by 3 h of incubation with HG in cardiomyocytes isolated from SGLT1 WT (n = 6), SGLT1 WT submitted to glu/gal free diet (n = 6) and SGLT1 KO (n = 6) mice. Data are means ± SEM. Statistical analysis was by two-way ANOVA. ^$^Indicates values statistically different from LG, p ≤ 0.05.

Next, we tested a SGLT1 knockout (KO) mouse model to evaluate SGLT1 involvement in HG-induced NOX2 activation. We first verified that SGLT1 KO mice exhibited a normal cardiac phenotype with normal left ventricular end-diastolic volume (LVEDV) (Fig. 5A), ejection fraction (EF) (Fig. 5B) and mass (Fig. 5C), under basal conditions. A glucose-galactose free diet, needed for SGLT1 KO mice survival, did not impact LV function (Fig. 5A–C). We also verified that SGLT1 deletion was not compensated by the expression of other SGLT isoforms in the heart (Fig. 5D) and did not affect NOX2 protein (gp91^phox^ and p47^phox^) expression (Supplementary Fig. 1). Cardiomyocytes of SGLT1 KO and wild-type (WT) mice were then isolated, cultured and exposed to HG. SGLT1 deletion did not prevent p47^phox^ translocation to cav3 (Fig. 5E) or ROS production (Fig. 5F) in response to HG, confirming the lack of implication of SGLT1 in cardiac NOX2 activation by HG. It should be noted that glucose/galactose free diet type did not affect HG-induced ROS production although it induced a slight, but not significant, reduction in p47phox translocation after HG (Fig. 5E,F).

### SMIT1 mediates HG-induced NOX2 activation and ROS production

**Figure 6:**
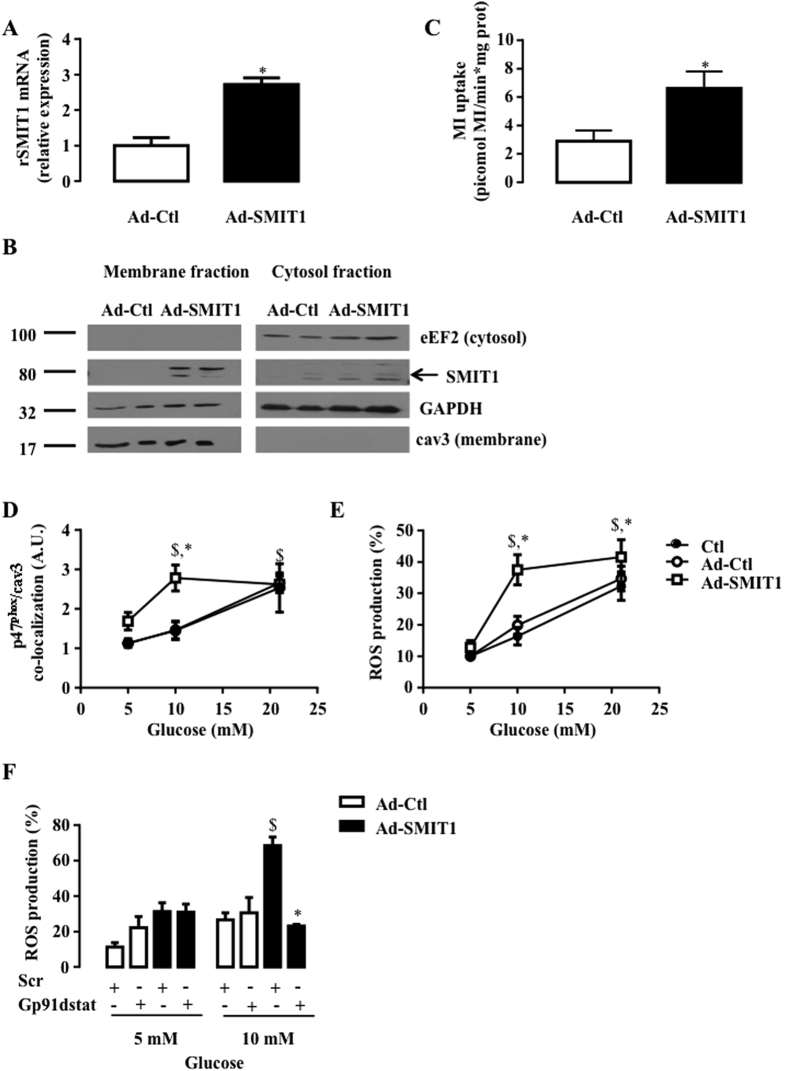
Impact of SMIT1 overexpression on NOX2 activation and ROS production. Adult rat cardiomyocytes were infected with adenoviruses (24 h, 200 MOI) expressing SMIT1 (Ad-SMIT1) or β-galactosidase (Ad-Ctl). (**A**) SMIT1 mRNA level measured by qRT-PCR (n = 3). Data were normalized to HPRT1 and expressed as relative expression vs Ad-Ctl. (**B**) SMIT1 protein expression in plasma membrane fractions obtained after cellular fractionation. Full-length blots are presented in Supplementary Figure 6. (**C**) Quantification of picomoles myo-[3 H]inositol uptake per min and mg of proteins (n = 4). (**D**) p47phox translocation close to cav3 (n = 6) and (**E**) ROS production (n = 7) in response to increased glucose concentration (5–10 and 21 mM of glucose). (**F**) Gp91dstat and scrambled peptide were added 15 min prior to glucose (5 or 10 mM glucose). ROS production was quantified 2 h after change in glucose concentration (n = 3). Data are means ± SEM. Statistical analysis was by (**A–C**) Student’s t-test or (**D,E,F**) two-way ANOVA. ^$^Indicates values statistically different from LG, p ≤ 0.05. *Indicates values statistically different from (**A–E**) Ad-Ctl, and (**F**) scr, p ≤ 0.05.

We resorted to genetic manipulation to evaluate the involvement of SMIT1 in HG-induced activation of NOX2 and ROS production. First, SMIT1 was overexpressed in adult rat cardiomyocytes with adenoviruses. Adenoviral infection induced a 3-fold increase of SMIT1 expression (Fig. 6A). Heightened SMIT1 protein expression was detected in plasma membrane fractions (Fig. 6B) and resulted in a significant 3-fold increment of myo-[^3^H]-inositol uptake (Fig. 6C). Under basal conditions or after infection with control adenovirus (Ad-Ctl), NOX2 activation (p47^phox^ translocation and ROS production) mediated by increased glucose concentration in culture medium, was dose-dependent, being maximal at 21 mM glucose (Fig. 6D,E). However, after SMIT1 overexpression, maximally-augmented p47^phox^ translocation to cav3 (Fig. 6D) and ROS production (Fig. 6E) were already observed with only 10 mM glucose, indicating that cardiomyocytes overexpressing SMIT1 were sensitized to glucose. In agreement, NOX2 inhibition, using 2.5 μM Gp91dstat, blunted SMIT1 overexpression-induced ROS production at 10 mM glucose (Fig. 6F).

**Figure 7:**
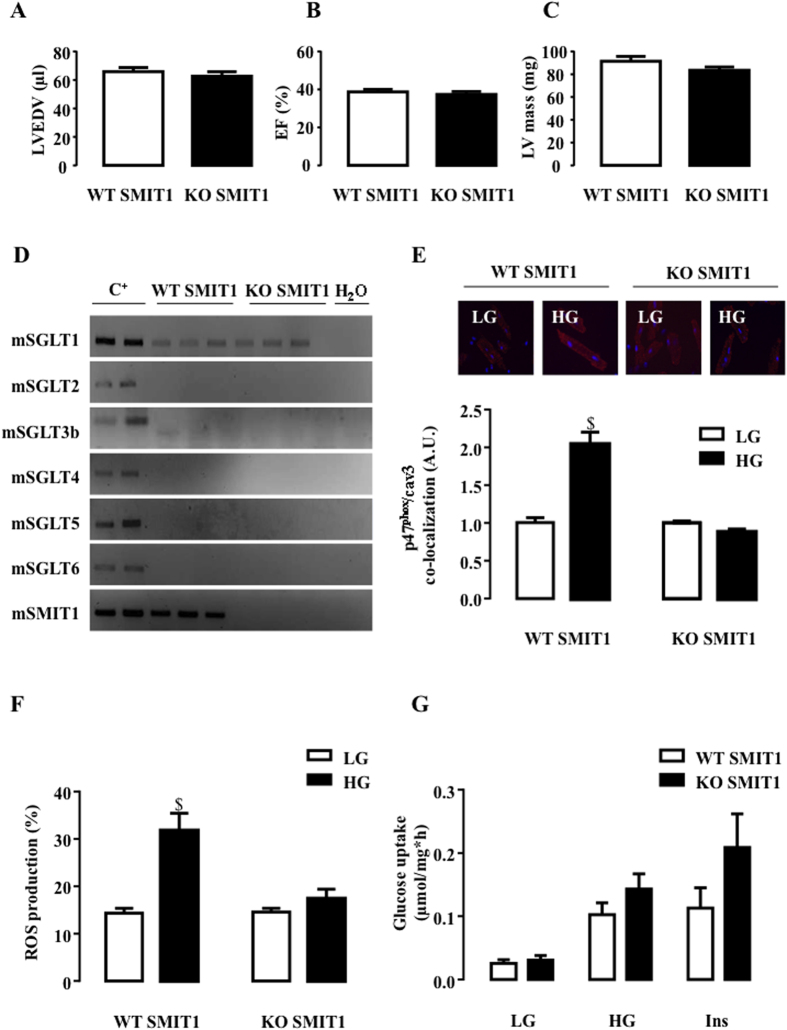
Impact of SMIT1 deletion on HG-induced NOX2 activation and ROS production. (**A**) LVEDV, (**B**) EF and (**C**) LV mass were measured by echocardiography of SMIT1 WT (n = 10) and KO (n = 10) mice. Echocardiographic data in M-mode and 2D parasternal long axis are presented in Supplementary Table 5. (**D**) Detection of SGLT1, SGLT2, SGLT3b, SGLT4, SGLT5 and SGLT6 by RT-PCR and ethidium bromide-stained agarose gels on mRNA extracted from the hearts of SMIT1 KO mice (n = 3) compared to WT mice (n = 3). Positive controls were intestine for SGLT1, kidneys for SGLT2, SGLT3, SGLT4 and SGLT5, brain for SGLT6 and SMIT1. (**E**) Quantification of HG-induced p47phox translocation close to cav3 in SMIT1 WT mice compared to SMIT1 KO mice. Adult mouse cardiomyocytes were isolated from SMIT1 WT (n = 4) or SMIT1 KO (n = 4) hearts. PLA was performed 90 min after stimulation with HG and compared to 5 mM of glucose. White lines correspond to 20 μm. (**F**) ROS production induced by 3 h of incubation with HG in cardiomyocytes isolated from SMIT1 WT (n = 6) or SMIT1 KO (n = 6) mice. (**G**) Cardiac glucose uptake in SMIT1 WT (n = 6) vs KO (n = 6) mice was measured under LG, HG and after insulin (3.10^−9^ M insulin 30 min). Data are means ± SEM. Statistical analysis was by two-way ANOVA (**E–F**). ^$^Indicates values statistically different from LG, p ≤ 0.05.

SMIT1 KO mice were used to provide a definitive proof of our new paradigm. As with SGLT1, the absence of SMIT1 affected neither the cardiac phenotype (Fig. 7A–C), the expression profile of other SGLT isoforms (Fig. 7D), nor NOX2 protein (gp91^phox^ and p47^phox^) expression (Supplementary Fig. 2). However, it drastically protected cardiomyocytes against hyperglycemic stress. Indeed, p47^phox^ was not translocated close to cav3 (Fig. 7E) and we did not observe an increase in ROS production (Fig. 7F) in SMIT1 KO cardiomyocytes in response to HG, in contrast to WT. The latter results definitively highlighted the role of SMIT1 in detecting high-glucose concentrations and in mediating ROS production in the heart.

As stated in the introduction section, we do argue that HG-induced NOX2 activation is not related to enhanced glycolysis and/or glucose metabolism. However, we had to verify that SMIT1 deletion did not interfere with cardiac glucose metabolism and more particularly that the inhibition of HG-induced ROS production in KO cells could not be attributed to a drastic reduction in glucose entry under HG. The rate of [2-^3^H] glucose uptake, a usual way to evaluate global glucose uptake, was measured in SMIT1 KO mice in comparison to WT at 5 (LG) and 21 mM glucose (HG) and in response to 3*10^−9^M of insulin. The absence of SMIT1 did not reduce glucose uptake under LG, HG or insulin stimulation (Fig. 7G). In contrast, a slight but non-significant increase in insulin response was observed. Similar data were obtained for 2[^3^H]-Deoxy-D-glucose uptake, another experimental procedure to evaluate glucose entry via GLUT transporters. Once again, these observations reinforce our hypothesis and data, disclosing that HG-induced ROS production is not related to changes in glucose metabolism.

## Discussion

The major findings of this study are that (i) SMIT1 is expressed as much as SGLT1 in the heart and (ii) detects elevated glucose concentration, leading to NOX2 activation and ROS production. It could therefore participate in redox signalling in normal and diabetic hearts. To the best of our knowledge, this is the first evidence of SMIT1’s role in cardiac tissue.

The SGLT2 isoform in the SGLT family has been widely explored in the literature because of its growing interest as a new therapeutic target in the treatment of type 2 diabetes (T2D). Indeed, SGLT2 inhibitors (SGLT2i) reduce plasma glucose levels by inhibiting glucose reabsorption, without targeting the major pathophysiological defects in T2D (insulin resistance and impaired insulin secretion). Interestingly, the EMPA-REG OUTCOME Trial recently showed that treatment of T2D patients at high risk for cardiovascular events with empagliflozin (the most selective SGLT2i) reduced major cardiovascular events, including death from cardiovascular causes, compared to placebo^35^. However, conflicting results still persist regarding its expression in the heart^16^,^26^,^29^. In the present study, we confirmed that SGLT2 is expressed neither in the cardiac tissue, nor in isolated cardiomyocytes, excluding a direct action of SGLT2i on heart. The cardiovascular protection conferred by empagliflozin could be due to increased salt excretion and decreased blood pressure. Identifying the role of SGLT2 in the vasculature requires further investigation.

In contrast to SGLT2, we confirmed that SGLT1 is highly expressed in the heart in agreement with earlier studies^26^,^27^. Our initial hypothesis was that SGLT1 sensed increased glucose concentrations, leading to ROS production, independently of glucose metabolism^11^. However, the high affinity of SGLT1 for glucose (Km = 0.5 mM) makes this paradigm unlikely. Theoretically, glucose transport through SGLT1 should be maximal under normoglycemia, protecting the cells against HG, unless there is a change in its expression or translocation. Our results definitely ruled out a role of SGLT1 in NOX2-dependent ROS production in the heart. On the one hand, galactose, a SGLT1 substrate, did not reproduce HG-induced NOX2 activation. Slightly increased ROS production due to galactose has been observed, although the latter was not sensitive to the NOX2 inhibitor. On the other hand, SGLT1 deficiency did not prevent cardiomyocytes from producing ROS in response to HG. Moreover, we showed that SGLT1 deletion did not impact LV function under basal conditions. Recent findings showed that SGLT1 protein is actually rather localized in the heart capillaries than in myocytes sarcolemma^36^. Taken all together, one may speculate that dual SGLT2/SGLT1 inhibitor should have limited cardiac side effects although they would be more effective in reducing glycemia^37^.

SMIT1, the third member of the Na^+^/glucose cotransporter family (*SLC5a3*), has been mainly studied in the brain where its function is to transport myo-inositol, an important precursor of inositol phosphates and phospholipids that are central in membrane and cell signalling^33^. We demonstrated, for the first time, that SMIT1 is expressed in mouse and rat hearts and cardiomyocytes, as well as in human hearts. SMIT1 expression data in human hearts come from patients with significant mitral disease without evidence of severe LV dilatation or dysfunction, referred for surgery (mitral valve plasty or replacement). We also verified that SMIT1 expression was similar in patients without any cardiovascular disease (heart rejected from transplant) (Supplementary Fig. 3). Compelling evidence favoured SMIT1 as HG sensor in the heart. Myo-inositol completely reproduced the toxic effects of HG, leading to NOX2 activation and ROS production. Furthermore, SMIT1 overexpression sensitized cardiomyocytes to glucose. Indeed, SMIT1 overexpression induced NOX2 activation and ROS production at only 10 mM of glucose, being nearly maximal. Finally, SMIT1 deletion prevented HG-induced NOX2 activation and ROS production.

Since SMIT1 deletion has no impact on cardiac phenotype, its physiological role in the heart remains to be elucidated. As in the brain, it could be involved in several transduction signals that have still to be determined. Interestingly, SMIT1 barely influenced glucose uptake in the heart in normoglycemia as well as in hyperglycemic conditions. Therefore, NOX2 activation could not be related to enhanced glucose uptake in HG but probably depends on a signalling cascade activated downstream of SMIT1. In line with this hypothesis, growing evidence favours SGLT transporters as glucose sensors beyond their role as active glucose transporters, triggering ionic signalling (Na^+^ and Ca2+ 14,31,38, via the sodium-calcium exchanger (NCX)^39^,^40^) into the cells owing to changes in extracellular glucose concentration^28^,^30^,^39^,^41^,^42^. Protein kinase C (PKC)-β, a calcium-dependent serine/threonine kinase, could be the link between ionic changes downstream of SMIT1 and NOX2 activation, as its inhibition prevented HG-induced ROS production^34^.

NOX2 seems to be the main source of ROS in our experimental model as the NOX2 inhibitor, gp91dstat, inhibited almost completely the HG-induced ROS production (Fig. 4C and ref. 11). However, one may not exclude that in the long term mitochondria also contribute to ROS production under hyperglycemic conditions. Indeed, NOX2 activation could trigger or enhance a subsequent mitochondrial ROS production^43^.

The pathophysiological role of acute HG-induced ROS production in the heart remains to be ascertained. Increased ROS production induces cell death in isolated cardiomyocytes in culture^11^ and is usually considered to be a key element contributing to the onset of diabetic cardiomyopathy and favouring heart failure. HG-induced ROS also triggers insulin resistance in cardiomyocytes. Indeed, incubation of cardiomyocytes with HG resulted in insulin resistance within 24 h, whereas NOX2 inhibition restored insulin signalling^11^. However, as previously advocated, cardiac insulin resistance state protects the heart from fuel overload in dysregulated metabolic states^44^. Therefore, NOX2-dependent ROS production, downstream of SMIT1, could be an “acute” intermediate signal, favouring anti-oxidant responses, inducing metabolic preconditioning and protecting cells against further fuel overload, including HG.

## Conclusion

In the present study, we highlighted that, besides SGLT1, SMIT1 is expressed in the heart. SMIT1 is able to detect increased extracellular glucose levels and triggers signalling leading to NOX2 activation and ROS production. To the best of our knowledge, ours is the first study which examines SMIT1 in the heart and proposes that it acts as a glucose sensor. Further investigation is required to ascertain its physiological and pathological functions.

## Methods

### Animals

Animal handling was approved by the Animal Research Committee of the Université catholique de Louvain (2012/UCL/MD/003) and conformed to the *Guide for the Care and Use of Laboratory Animals* published by the US National Institutes of Health (NIH Publication No. 85–23, revised 1996). SGLT1 KO mice and SMIT1 KO mice were generated as described elsewhere^14^,^45^.

### Materials

D-(+)-glucose (G8272), methyl-α-D-glucopyranoside (M9376), D-(+)-mannose (M6020), myo-inositol (I5125), D-(+)-galactose (G0750), 2-deoxy-D-glucose (D3179) and 1-deoxy-D-glucose (S404497) were purchased from Sigma. The antibodies used were: SMIT1 (C18843) from Assay BioTech and (Ab113245) from Abcam, Caveolin-3 (sc-7665) from Santa Cruz Biotechnology, p47^phox^ (07–497) from Millipore, and gp91^phox^ (Sc-5827) from Santa Cruz. Gp91dstat peptide and corresponding scrambled peptide were kindly provided by V. Stroobant (Ludwig Institute, Brussels, Belgium).

### Isolation and culture of adult rat and mice cardiomyocytes

Adult rat cardiomyocytes were isolated after heart perfusion with collagenase type II (1 mg/ml, Worthington), then purified and cultured, as described previously^46^. Primary cultures of cardiomyocytes were incubated with different sugar concentrations for the indicated periods of time, as detailed in the figure legends.

Adult mice were anesthetized with a mixture of ketamine (80 mg/kg) and xylazine (10 mg/kg). Cardiomyocytes were isolated, as described^34^, with the following modifications: hearts were perfused with Liberase DH enzyme (0.625 mg/heart, Roche) and trypsin (2.1 mg/heart, Life Technologies 15090–046). The cells were then purified, cultured and plated on laminin-coated dishes, and incubated for 1 h in fresh minimum essential medium (MEM) with Hank’s salts (Life Technologies 11575–032) supplemented with L-glutamine (2 mM), bovine serum albumin (BSA) 100 μg/ml, penicillin (100 U/ml) and streptomycin (100 μg/ml). Primary cultures of mice cardiomyocytes were treated as adult rat cardiomyocytes.

### Human biopsies

Human LV biopsies were obtained during surgery from patients operated because of severe mitral disease but without any evidence of LV dilatation or dysfunction (baseline clinical characteristics of patients are presented in Supplementary Tables). This protocol was approved by the local ethics committee (Comité éthique hospitalo-facultaire des Cliniques Universitaires St. Luc, Brussels, Belgium) and signed informed consent was obtained before surgery. All experimental procedures were performed in accordance with relevant guidelines and regulations. All cardiac tissues were immediately frozen in liquid nitrogen and stored at −80 °C. Brain, intestine and kidney samples, as positive controls, were residual material from surgery.

### RNA extraction and RT-PCR

Total mRNA was extracted from organs with an RNA extraction kit (RNeasy mini-kit Qiagen 74106) and from cardiomyocytes according to a chloroform/isopropanol procedure (Tripure Isolation reagent, Roche 11667165001). All RNA preparations were subjected to on-column treatment with RNAse-free DNAse set (Qiagen 79254). mRNAs were quantified with nanodrop (Thermo Scientific). Reverse transcription was performed for 1 h at 37 °C with 0.4 to 1 μg RNA and iScript^TM^ cDNA synthesis kit (Bio-Rad laboratories 1708891). All PCRs contained 5 μl of RT reaction and 0.05 μM of forward and reverse primers in 50 μl volume. PCR was performed with GoTaq G2 DNA polymerase (Promega M7845), and reactions were heated to 95 °C for 2 min, followed by up to 45 cycles of 95 °C for 30 s, 60–65 °C for 30 s, 72 °C for 40 s and then heated at 72 °C for additional 5 min in a MJ Mini^TM^ Gradient Thermal Cycler (Biorad PTC-1148). The PCR products were separated by agarose gel electrophoresis (0.8 to 2% wt/v) and stained with ethidium bromide. All PCR products were verified by sequencing (Beckman Coulter Genomics). Nucleotide sequences of the primers used are summarized in Supplementary Tables. qRT-PCR was performed on IQ5 (Bio-Rad) with qPCR Core kit from Sybergreen (Eurogentec RT-SN-10–05-NR). The mRNA level for each gene in each sample was normalized to a housekeeping gene. Standards consisted of 10-fold dilutions of plasmids carrying the PCR fragment of interest (pcDNA3-Topo, Invitrogen). The nucleotide sequences of primers used are summarized in Supplementary Tables.

### ROS measurement

Intracellular ROS production was measured, as described^11^ previously, by evaluating oxidation of the cell permeable fluorescent probe 2′,7′-dihydrodichlorofluorescein diacetate (Invitrogen), which becomes fluorescent when oxidized by ROS.

### Preparation of cytosol and membrane fractions

Cytosol and membrane fractions were obtained, as described previously^47^. After appropriate stimulation, cardiomyocytes were washed 3 times in ice-cold Dubbelcco’s phosphate-buffered saline (PBS) and scraped in buffer A containing 20 mM Tris pH 7.5, 2.5 mM EGTA, 0.1 mM EDTA, 0.1 M NaF, 2 mM dithiothreitol and protease inhibitor cocktail (cOmplete Mini from Roche). Cells were disrupted by sonication on ice (five 4-s bursts) and then centrifuged at 1,500 *g* for 10 min at 4 °C. Supernatants were collected (cytosolic fraction) and centrifuged at 100 000 g for 45 min at 4 °C. The pellets were solubilized in buffer A containing 1% triton X-100, sonicated and centrifuged at 15 000 *g* for 15 min at 4 °C. The resulting supernatants were membrane fractions.

### Immunoblotting analysis

Protein content was measured by the Bradford method with BSA as standard. Immunoblotting was performed with extracts separated on 8% (SMIT1, p47^phox^, gp91^phox^) or 12% (Cav3) SDS-PAGE and transferred to polyvinylidene difluoride membranes which were blocked with BSA (5% wt/v) and then with corresponding antibodies. After incubation with appropriate secondary antibody (anti-rabbit and anti-goat), proteins were visualized by electrochemical luminescence (Pierce).

### Protein co-localization by *in situ* proximity ligation assay (PLA)

p47^phox^ co-localization with cav3 was measured, as described^34^ previously and visualized as red fluorescent dots appearing where both proteins co-localize. Nuclei were stained with DAPI. Protein staining was visualized with a Zeiss.Imager.Z1 microscope equipped with an ApoTome device. Red fluorescent dots were quantified by AxioVision Rel 4.8 software.

### SMIT1 adenovirus generation and cardiomyocytes infection

SMIT1 adenoviruses were generated with the Adeasy system (Agilent Technologies). Briefly, SMIT1 cDNA was amplified by PCR from rat brains with the following primers: sense 5′-ATGAGGGCTGTGCTGGAGAC-3 and antisense 5′-TCATAAGGAGAAATAAACAAACAT-3′, and inserted into pShuttle-CMV vector. pShuttle-SMIT1 was recombined in pAdEasy vector. Adenovirus production and amplification were realized as per the manufacturer’s instructions.

Adult rat cardiomyocytes were infected (200 multiplicity of infection) with adenoviral construction (Ad-SMIT1 and β-galactosidase adenoviruses served as control: (Ad-Ctl). SMIT1 expression was assessed by qRT-PCR, cell fractionation and myo-inositol uptake, 24 h after cardiomyocyte infection.

### Myo-inositol uptake

100,000 cells were infected (Ad-SMIT1 or Ad-Ctl) for 24 h, then rinsed with PBS and incubated for 15 min at 37 °C in medium containing 1.2 mM MgSO_4,_ 1.2 mM KH_2_PO_4_, 4.7 mM KCl, 120 mM NaCl, 2.5 mM CaCl_2_ and 25 mM NaHCO_3_ in 5% CO_2_ at pH 7.4. This medium was replaced by the same solution containing 25 μM MI (0.08 μCi/1μmol) for 1 min at 37 °C. The cells were then rinsed twice with the same solution containing 3 mM phlorizin and scrapped in 100 mM NaOH. Myo-inositol uptake was linear from 30 s to 5 min.

### Glucose uptake

Glucose uptake was measured as described previously^46^,^48^, by [2-^3^H]glucose detritiation rate which occurs after glucose phosphorylation during rapid isomerization of hexose-6-phosphate catalyzed by phosphoglucose isomerase. Tritium from C2 of glucose is released as tritiated water in supernatant, which can be separated from tritiated glucose by chromatography on anion exchange resin (borate form) and measured by scintillation counter.

### Echocardiography

Two-dimensional echocardiography was performed on mice with a Vevo 2100 Imaging system (VisualSonics), as described previously^49^. Mice were anesthetized by isoflurane inhalation at concentrations of 5% (induction) and 2,5% (maintenance) in 100% oxygen.

### Statistical analysis

All values are expressed as means ± SEM. Samples are biological replicates. Comparisons were made by ANOVA, followed by Bonferroni post hoc testing (detailed in the figure legends). p < 0.05 values were considered to be statistically significant.

## Additional Information

**How to cite this article:** Van Steenbergen, A. *et al*. Sodium-myoinositol cotransporter-1, SMIT1, mediates the production of reactive oxygen species induced by hyperglycemia in the heart. *Sci. Rep.* **7**, 41166; doi: 10.1038/srep41166 (2017).

**Publisher's note:** Springer Nature remains neutral with regard to jurisdictional claims in published maps and institutional affiliations.

## Supplementary Material

Supplementary Information
