# Supplementary material for: Sodium-myoinositol cotransporter-1, SMIT1, mediates the production of reactive oxygen species induced by hyperglycemia in the heart
Source: Sci Rep. 2017 Jan 27;7:41166. doi: 10.1038/srep41166 (PMC5269587; doi:10.1038/srep41166)

Title:

Sodium-myoinositol cotransporter-1, SMIT1, mediates the production of reactive oxygen species induced by hyperglycemia in the heart

Authors:

Anne Van Steenbergen, Magali Balteau, Audrey Ginion, Laura Ferté, Sylvain Battault, Christophe de Meester de Ravenstein, Jean-Luc Balligand, Evangelos-Panagiotis Daskalopoulos, Patrick Gilon, Florin Despa, Sanda Despa, Jean-Louis Vanoverschelde, Sandrine Horman, Hermann Koepsell, Gerard Berry, Louis Hue, Luc Bertrand, Christophe Beauloye

Supplementary table 1: Patients' characteristics

**Basic clinical characteristics**	**Study population**
	**n=7**
Age, yr	62 ± 9
Male Gender, n(%)	5 (71)
BMI, kg/m^2	28 ± 5
Active smoking, n (%)	0 (0)
Diabetes, n (%)	1 (14)
Family history of MI, n (%)	1 (14)
Hypertension, n (%)	3 (43)
Hypercholesterolemia, n (%)	1 (14)
LVEDV (ml)	205 ±74
LVESV (ml)	86 ± 31
EF, %	59 ± 7
Left ventricular mass (g)	142 ± 37

Supplementary table 2: Primer sequences and PCR conditions

Amplified genes	Sense (S) / antisense (AS)	Rats	Mice	Humans	Annealing temp. (°C)
**SGLT1**	S	TCTTCGCTATCAGCGTCGTC	GCCATCATCCTCTTCGTCAT	TGCTGGTGGGGTCTTTAATC	57;64.4;59
	AS	TGCGCTCTTCTGTGCTGTTA	TTGGAGTCCTCTGGGATGTC	GCCAACCTTGGTACCGCAAT	
**SGLT2**	S	CGCCATCATTCTCTTCTTCTGCT	ATTGTCTCGGGCTGGTATTG	TCCTCACCCTCACGGTCTC	61;59;59
	AS	CCTGCCGTATTTTTGCCCTTTT	TTAGAGCAGCCCACCTCAGT	CTGGGGCTCATTCATCTCCA	
**SGLT3**	S	GAACATGTCCCACGTGAAGGC	CTGTTGGAGGCTTCTTCCTG	CTCGCTGGTCGTGATATGGCC	57;60;64.3
	AS	TGCAGAAGATGGCAAGCAAGAAC	GGAGAAACACCCAACCAAGA	GCCAACATCAACGCCACAGTG	
	Nested-S	ATGCTGTCGGTCATGTTGGC	-	-	
	Nested-AS	GATGTAGTGGAAGAGCTGTCC	-	-	
**SGLT4**	S	ACCTGTCACCTCCCACGG	CCTGGTGGACAAGGAACTGT	CCCTTGGCCAGGTCTCATTT	57;59;59
	AS	ATATTGGAGCATCCAACTCTGGC	GAACCAGTTCCACAGCCATT	CCCACTGTTGGAGCTTTGGA	
	Nested-S	ATGCCTTCCACATGCTTCGAG	-	-	
	Nested-AS	TGACAGATGTCAGGGTCCAC	-	-	
**SGLT5**	S	TTTTGCCTTGAATGTGGCCG	GATCGGAGCCTCACTCTTTG	TACGCGGGGGCTCTGTTTGT	65;59;59
	AS	GCTCGTAGACACTCGGATGG	GCATCAGGGACAGGACAGAT	GGGAGCTGGTCACTGACTGCAT	
**SGLT6**	S	TGATTGTGGGCAGGGTGTTT	CCCAAACTTGTGCTGGAACT	CGGTCCTGGTGAAGAGCATT	61.2;59;59
	AS	CGTGATGAGGGTGGTAGACG	GATCCAGAGGATGGAGACCA	GGCATCCCGTTCTGAGAGAG	
**SMIT1**	S	CCAGCCTCGGTATGGTACTG	CGTGGCTACGGCATTATTTT	GGGGTTGGTACAGTAGGCTTC	60.8;62.5;55.8
	AS	GCATCTCCACAATAATTGGC	CAGACTTCCCGTTGGGAATA	GGGCCACTATGGCAATGTCT	

Supplementary table 3: Primer sequences and qPCR conditions

Amplified genes	Sense (S) / antisense (AS)	Rats	Mice	Humans	Annealing temp. (°C)
**SGLT1**	S	TCTTCGCTATCAGCGTCGTC	GCCATCATCCTCTTCGTCAT	TGCTGGTGGGGTCTTTAATC	57;64.4;64.4
	AS	TGCGCTCTTCTGTGCTGTTA	TTGGAGTCCTCTGGGATGTC	GGATCTCGGAAGATGTGGAA	
**SMIT1**	S	TGGTGACGAAGGAGAGTTGC	CGTGGCTACGGCATTATTTT	GCTCATGCCAAAGGCTCTAC	62;62.5,64.4
	AS	GGTTGGAGCCCCTTAATGCT	CAGACTTCCCGTTGGGAATA	CACAGGAACCAGCTTCATCA	
**hPRT1**	S	GTCCCAGCGTCGTGATTAGT	-	-	60
	AS	ACAGAGGGCCACAATGTGAT	-	-	
**RPL32**	S	-	GGCACCAGTCAGACCGATAT	AGGCATTGACAACAGGGTTC	60;62.5
	AS	-	CAGGATCTGGCCCTTGAAC	GTTGCACATCAGCAGCACTT	

Supplementary table 4: Echocardiographic data of SGLT1$^{-/-}$ and $^{+/+}$ mice

Parameter	WT SGLT1	WT SGLT1 (glu-gal free diet)	KO SGLT1 (glu-gal free diet)
**M-mode**	(n=10)	(n=10)	(n=10)
End-diastolic interventricular septal thickness (mm)	0.61 ± 0.04	0.57 ± 0.03	0.56 ± 0.04
End-systolic interventricular septal thickness (mm)	0.82 ± 0.05	0.81 ± 0.05	0.78 ± 0.06
End-diastolic left ventricular internal diameter (mm)	4.32 ± 0.12	4.14 ± 0.09	4.14 ± 0.07
End-systolic left ventricular internal diameter (mm)	3.3 ± 0.13	3.16 ± 0.08	3.27 ± 0.09
End-diastolic left ventricular posterior wall thickness (mm)	0.66 ± 0.05	0.66 ± 0.03	0.59 ± 0.01
End-systolic left ventricular wall thickness (mm)	0.92 ± 0.05	0.91 ± 0.04	0.81 ± 0.02*
Fractional shortening (%)	23.82 ± 1.6	23.68 ± 1.7	21.16 ± 1.52
**2D-parasternal long axis**			
Left ventricular end-diastolic volume (µl)	72 ± 4	60 ± 4	62 ± 3
Left ventricular end-systolic volume (µl)	39 ± 2	34 ± 2	34 ± 2
Ejection fraction (%)	45 ± 1	44 ± 1	45 ± 2
Left ventricular mass (mg)	93 ± 5	85 ± 3	83 ± 2
Heart rate (BPM)	448 ± 12	399 ± 12	395 ± 9

*p<0.05 vs WT

Supplementary table 5: echocardiographic data of SMIT1$^{-/-}$ and $^{+/+}$ mice

Parameter	WT SMIT1	KO SMIT1
**M-mode**	(n=10)	(n=10)
End-diastolic interventricular septal thickness (mm)	0.58 ± 0.03	0.61 ± 0.03
End-systolic interventricular septal thickness (mm)	0.75 ± 0.04	0.84 ± 0.04
End-diastolic left ventricular internal diameter (mm)	4.18 ± 0.06	4.19 ± 0.11
End-systolic left ventricular internal diameter (mm)	3.43 ± 0.07	3.45 ± 0.12
End-diastolic left ventricular posterior wall thickness (mm)	0.62 ± 0.032	0.56 ± 0.02
End-systolic left ventricular wall thickness (mm)	0.80 ± 0.05	0.73 ± 0.03
Fractional shortening (%)	18.00 ± 1.2	16.76 ± 1.4
**2D-parasternal long axis**		
Left ventricular end-diastolic volume (µl)	66 ± 3	63 ± 3
Left ventricular end-systolic volume (µl)	40 ± 1	39 ± 2
Ejection fraction (%)	39 ± 1	37 ± 1
Left ventricular mass (mg)	91 ± 4	83 ± 3
Heart rate (BPM)	406 ± 12	397 ± 21

Supplementary Fig. 1

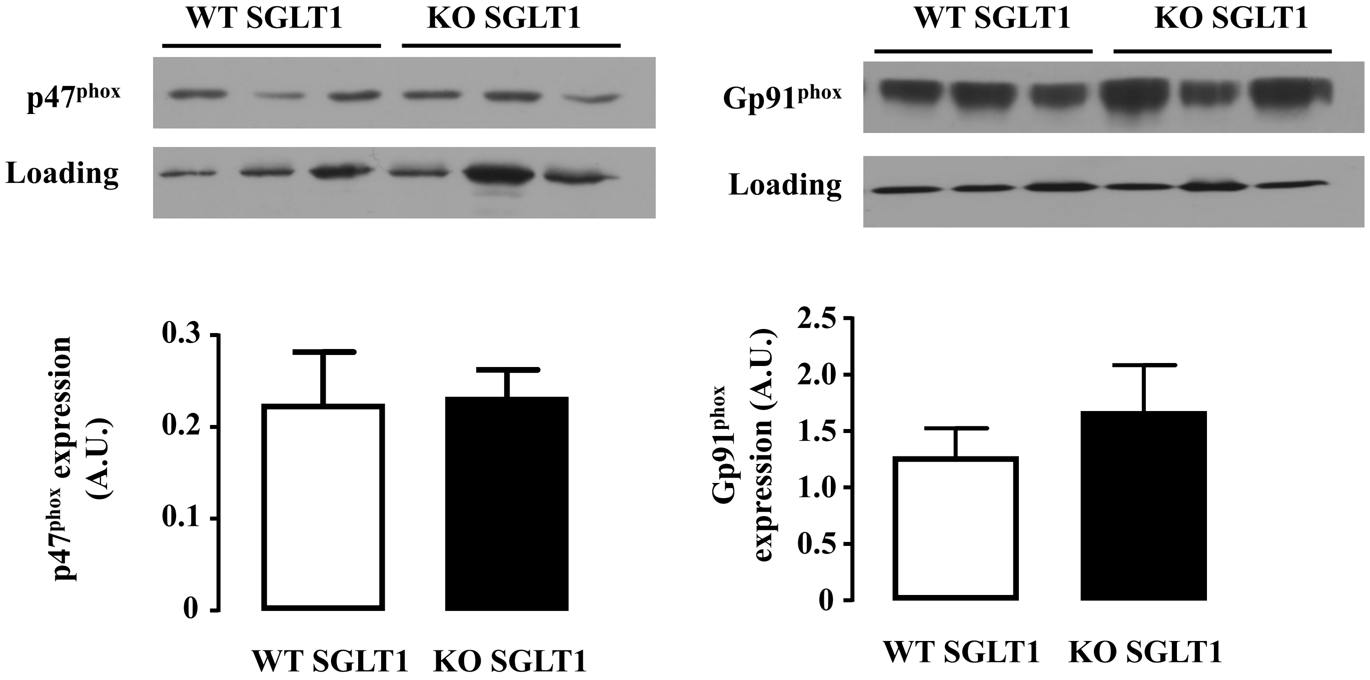

Supplementary Figure 1. NOX2 subunits protein expression in SGLT1-/- cardiomyocytes
Western blot analysis of $p47^{phox}$ and $gp91^{phox}$ on cardiomyocytes isolated from SGLT1 WT and KO mice. Loading control corresponds to GADPH. Blots and data quantification are presented. Data are means ± SEM. Statistical analysis was by Student's *t*-test.

Supplementary Fig. 2

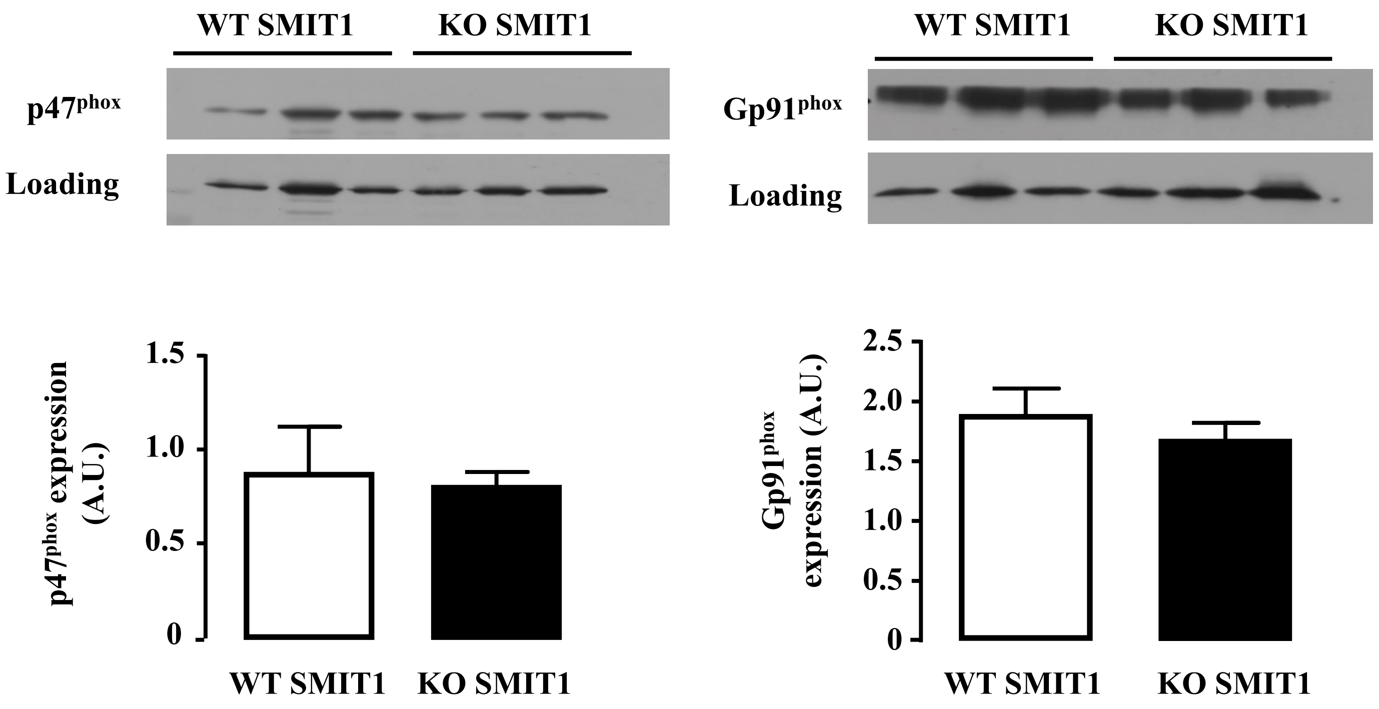

Supplementary Figure 2. NOX2 subunits protein expression in SMIT1-/- cardiomyocytes
Western blot analysis of $p47^{phox}$ and $gp91^{phox}$ on cardiomyocytes isolated from SMIT1 WT and KO mice. Loading control corresponds to GADPH. Blots and data quantification are presented. Data are means ± SEM. Statistical analysis was by Student's *t*-test.

Supplementary Fig. 3

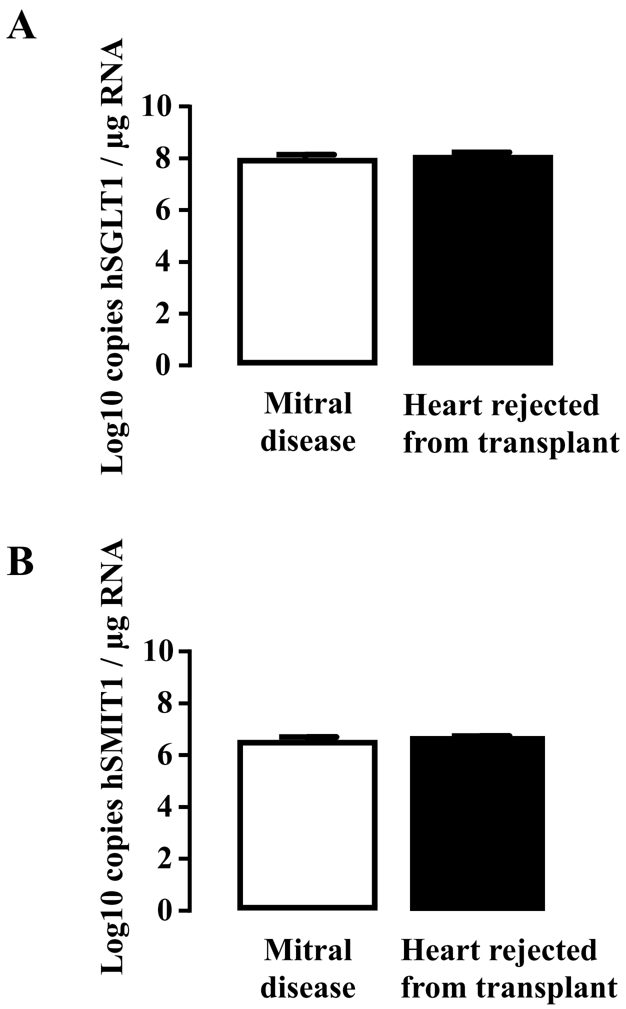

Supplementary Figure 3. Quantification of SMIT1 expression in human heart
mRNA copy numbers/µg of (A) SGLT1 or (B) SMIT1 RNA were measured in hearts from patients suffering from mitral disease (n=7) or from heart rejected from transplant (n=3). Data were normalized to RPL32 and expressed as Log10 copy numbers/µg RNA. Data are means ± SEM. Statistical analysis was by Student's *t*-test.

Supplementary Fig. 4 (blots from Fig. 1E)

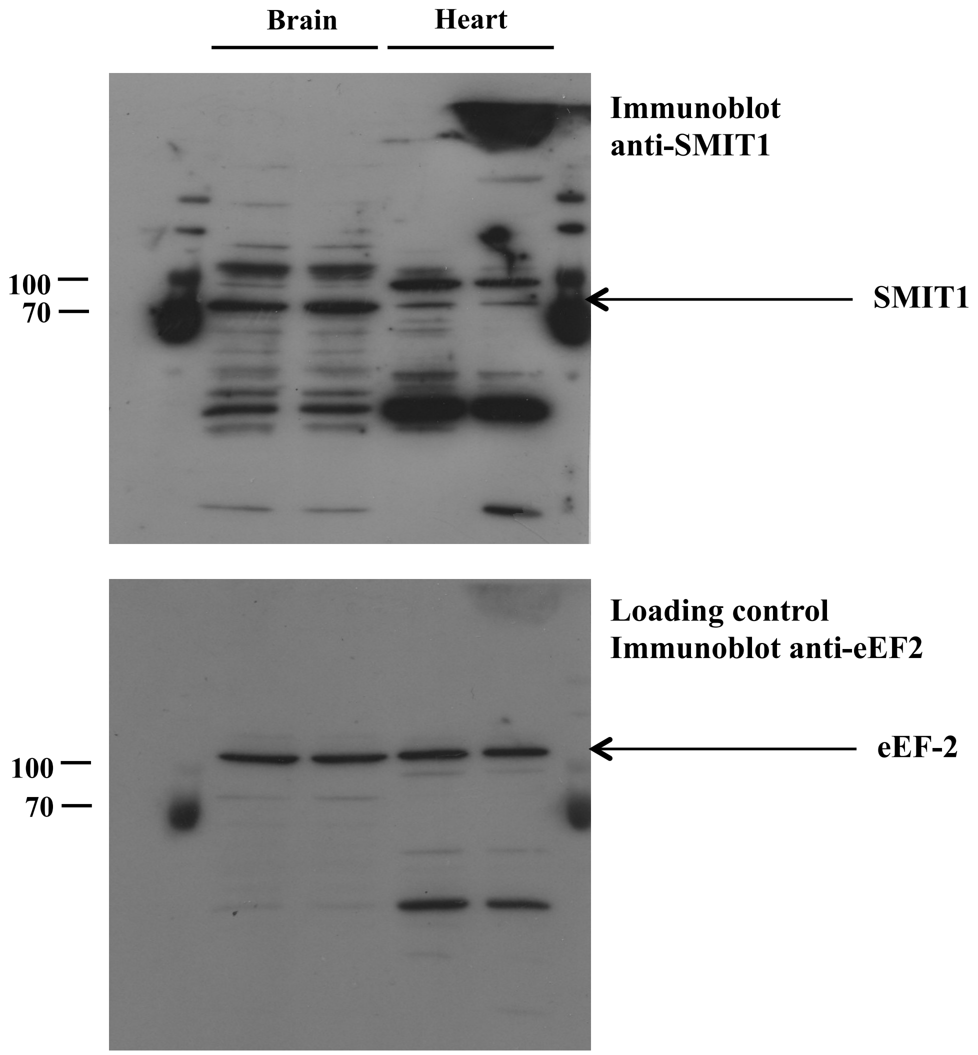

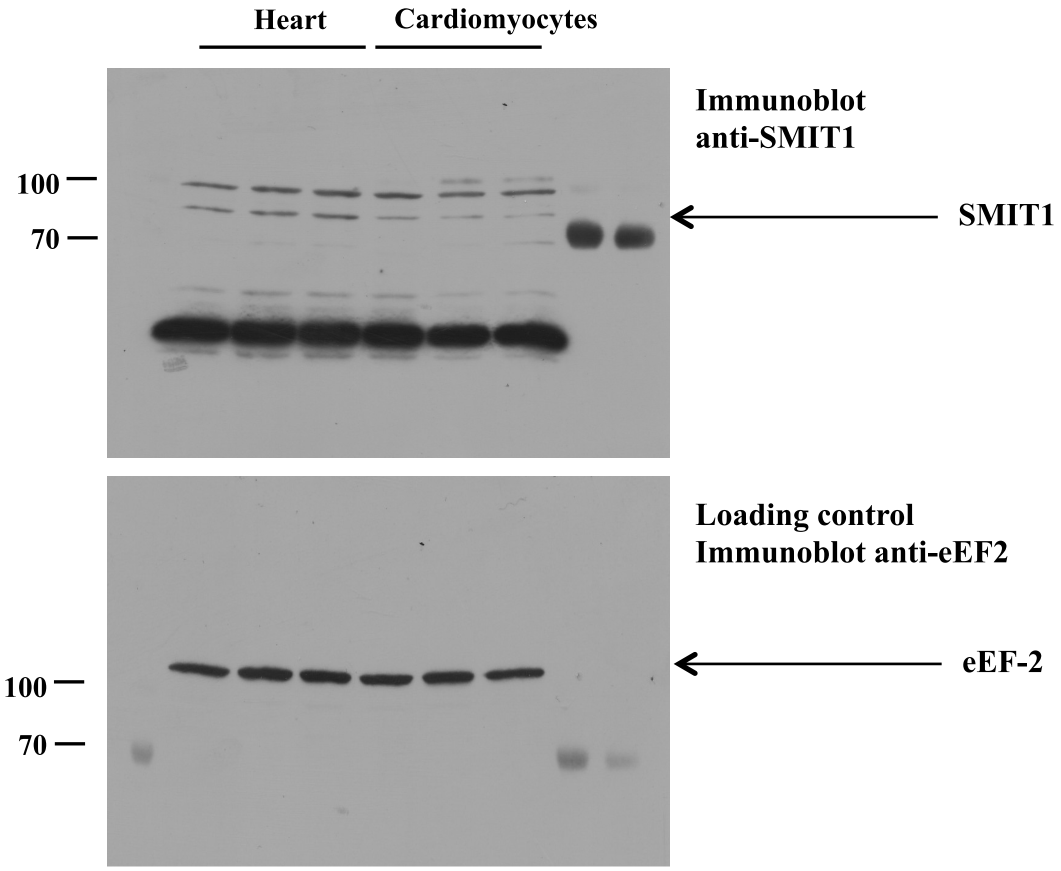

Supplementary Fig. 5 (blots from Fig. 2E)

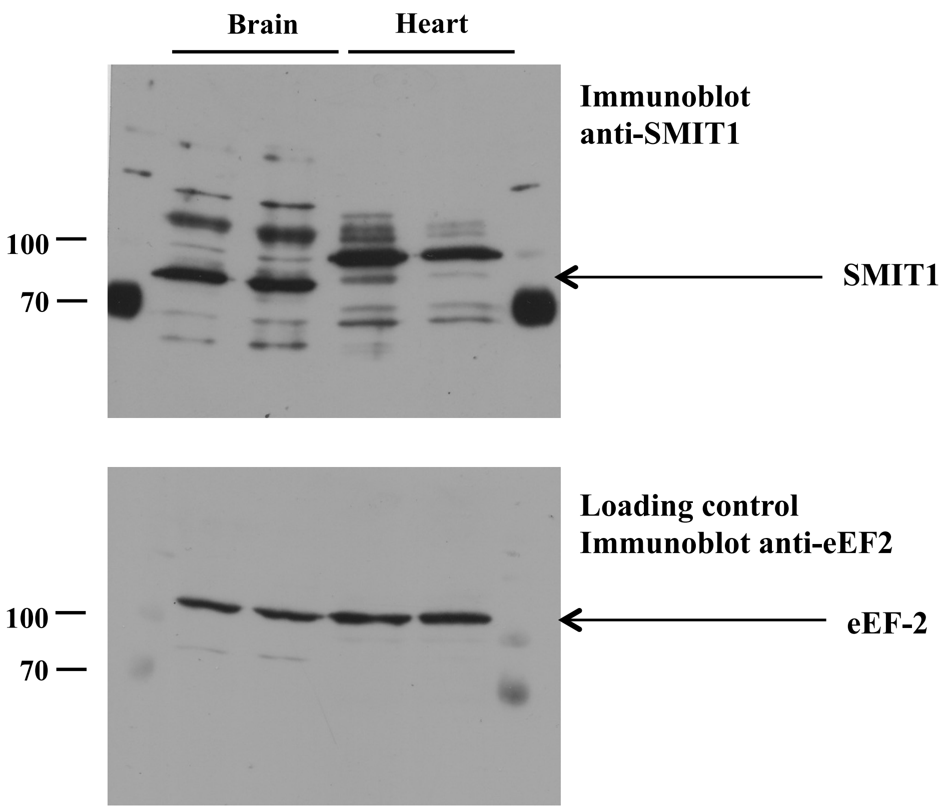

Supplementary Fig. 6 (blots from Fig. 6B)

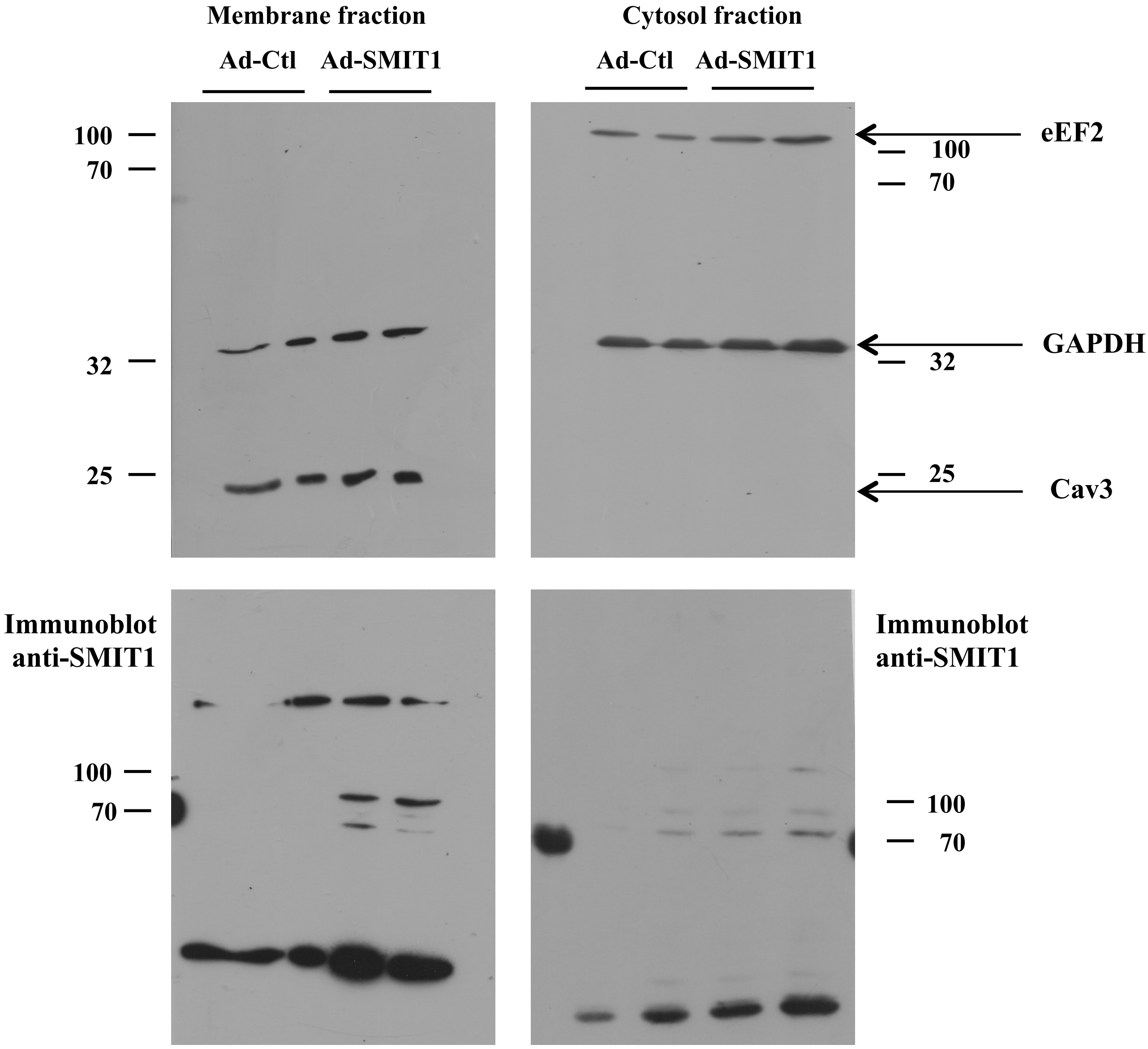

Supplement: Supplementary Information [file srep41166-s1.pdf]
